# Dexmedetomidine decreases the inflammatory response to myocardial surgery under mini-cardiopulmonary bypass

**DOI:** 10.1590/1414-431X20154646

**Published:** 2016-02-23

**Authors:** N.M.H. Bulow, E. Colpo, R.P. Pereira, E.F.M. Correa, E.P. Waczuk, M.F. Duarte, J.B.T. Rocha

**Affiliations:** 1 Departamento de Bioquímica e Biologia Molecular, Programa de Pós-graduação em Ciências Biológicas - Bioquímica Toxicológica, Centro de Ciências Naturais e Exatas, Universidade Federal de Santa Maria, Santa Maria, RS, Brasil; 2 Departamento de Cirurgia, Centro de Ciências da Saúde, Universidade Federal de Santa Maria, Santa Maria, RS, Brasil; 3 Departamento de Química, Programa de Pós-graduação em Química Aplicada, Universidade Estadual de Ponta Grossa, Ponta Grossa, PR, Brasil; 4 Departamento de Nutrição, Centro Universitário Franciscano, Santa Maria, RS, Brasil

**Keywords:** Cytokines, Systemic inflammatory response syndrome, Total intravenous anesthesia, Dexmedetomidine, Coronary artery bypass grafting surgery, Mini-cardiopulmonary bypass

## Abstract

Cardiopulmonary bypass (CPB) with extracorporeal circulation produces changes in the immune system accompanied by an increase in proinflammatory cytokines and a decrease in anti-inflammatory cytokines. We hypothesize that dexmedetomidine (DEX) as an anesthetic adjuvant modulates the inflammatory response after coronary artery bypass graft surgery with mini-CPB. In a prospective, randomized, blind study, 12 patients (4 females and 8 males, age range 42-72) were assigned to DEX group and compared with a conventional total intravenous anesthesia (TIVA) group of 11 patients (4 females and 7 males). The endpoints used to assess inflammatory and biochemical responses to mini-CPB were plasma interleukin (IL)-1, IL-6, IL-10, interferon (INF)-γ, tumor necrosis factor (TNF)-α, C-reactive protein, creatine phosphokinase, creatine phosphokinase-MB, cardiac troponin I, cortisol, and glucose levels. These variables were determined before anesthesia, 90 min after beginning CPB, 5 h after beginning CPB, and 24 h after the end of surgery. Endpoints of oxidative stress, including thiobarbituric acid reactive species and delta-aminolevulinate dehydratase activity in erythrocytes were also determined. DEX+TIVA use was associated with a significant reduction in IL-1, IL-6, TNF-α, and INF-γ (P<0.0001) levels compared with TIVA (two-way ANOVA). In contrast, the surgery-induced increase in thiobarbituric acid reactive species was higher in the DEX+TIVA group than in the TIVA group (P<0.01; two-way ANOVA). Delta-aminolevulinate dehydratase activity was decreased after CPB (P<0.001), but there was no difference between the two groups. DEX as an adjuvant in anesthesia reduced circulating IL-1, IL-6, TNF-α, and INF-γ levels after mini-CPB. These findings indicate an interesting anti-inflammatory effect of DEX, which should be studied in different types of surgical interventions.

## Introduction

Exacerbated immunoinflammatory responses to cardiopulmonary bypass (CPB) have been compared with systemic inflammatory response syndrome (SIRS) (1 2 3). Surgical trauma, cardiac arrest, aortic cannulation, exposure of blood to nonphysiological surfaces of the cardiopulmonary bypass circuit, and ischemia/reperfusion injury trigger the excessive release of proinflammatory factors (cytokines, endothelins, platelet-activating factors, and endothelial and leukocyte adhesion molecules) (4 5 6 7). An atypical inflammatory response observed in SIRS and after CPB has been reported to be associated with oxidative stress (4,8,9).

Anesthetic agents can exert clinical benefits during and after surgery (5,9 10 11 12 13). Optimistically, the ideal anti-inflammatory anesthetic should reduce complications and mortality resulting from SIRS-like postoperative responses. Alpha 2-adrenergic receptor agonists have been used in anesthesia because of their sedative, analgesic, hemodynamic-stabilizing, and sympatholytic effects (9,14,15). In addition, the stress response to surgery can be modulated by postsynaptic central α 2-adrenergic receptor activation (16). However, only a few studies have investigated the anti-inflammatory properties of dexmedetomidine (DEX) after coronary artery bypass graft (CABG) surgery with mini-CPB (9,16 17 18 19).

Safe and effective agents with anti-inflammatory properties to be used as anesthetics in major surgeries need to be identified (5,9). In this regard, DEX is considered as a promising candidate because α 2-adrenergic agonists have been reported to modulate inflammatory responses (13,16 17 18 19).

We hypothesize that DEX in association with a conventional total intravenous anesthesia (TIVA) (infusion of propofol and sufentanil) decreases the inflammatory response associated with CABG surgery. To test this hypothesis, we measured interleukin (IL)-1, IL-6, IL-10, tumor necrosis factor (TNF)-α, and interferon (IFN)-γ levels as endpoints of inflammation in patients undergoing CABG surgery. Because the inflammatory response can trigger oxidative stress, two biochemical endpoints of oxidative stress, thiobarbituric acid reactive substances (TBARS) (20) and delta-aminolevulinate dehydratase (ALA-D) activity (21), were also determined in erythrocytes.

## Material and Methods

Institutional ethics review board approval (Protocol #23081.015056/2009-58, Approval #0271.0.243.000-9, Research Ethical Committee of UFSM in January 2010) and written informed consent from patients were obtained. A total of 30 clinical American Society of Anesthesiologists class II and III patients (42-72 years old) were included in the study. They were scheduled for CABG surgery under mini-CPB and randomly assigned to the conventional TIVA (propofol/sufentanil) group (15 patients) or to the TIVA with DEX (propofol/sufentanil/DEX) (TIVA+DEX; 15 patients) group. The surgery team, surgeon, and perfusionist were the same for all of the patients. Patients were recruited during a period of 2 years during which data were collected.

Exclusion criteria included the following: severe ventricular dysfunction (left ventricular ejection fraction <40%), reintervention surgery, requirement for blood products from the start of CPB, preoperative history of liver or kidney dysfunction, immunological disease, preoperative intake of corticosteroids or anti-inflammatory drugs (except for acetylsalicylic acid), and a history of recent myocardial infarction (last 2 weeks).

Patients who were allocated to the TIVA group were anesthetized with TIVA in target-controlled infusion of propofol as a hypnotic (TCI infusion system, Diprifusor^¯^; AstraZeneca, Germany). Propofol was infused to an initial target blood concentration of 4 μg/mL during the induction and maintenance of anesthesia. The target dose was based on bispectral (BIS) index monitoring (i.e., the dose was selected to have BIS values between 45 and 55). Sufentanil was infused at a dose of 0.5 to 1 µg/kg (induction) and maintained at 0.5 to 1 µg·kg^-1^·h^-1^ during surgery. Muscle relaxation for tracheal intubation was obtained with 0.1 mg/kg pancuronium at induction and an additional one-third of this dose was provided if necessary.

Patients who were allocated to the TIVA+DEX group were anesthetized with TIVA in target-controlled infusion of propofol as a hypnotic (TCI infusion system, Diprifusor). The initial target blood concentration of propofol was 4 μg/mL for induction and maintenance of anesthesia. Sufentanil was administered at 0.5 to 1 µg/kg during induction and at rates of 0.5 to 1 µg·kg^-1^·h^-1^ during the maintenance period. DEX was infused at 0.3 µg·kg^-1^·h^-1^ during the entire surgery (induction and maintenance of anesthesia). The dose of DEX was lower than the average dose that is commonly used in surgeries to prevent bradycardia and because it was used together with sufentanil and propofol. Muscle relaxation for tracheal intubation was obtained with 0.1 mg/kg pancuronium at induction and an additional one-third of the dose was provided if necessary. Surgeons working in the operation room and the medical team in the intensive care unit (ICU) were blind to treatment protocols.

Systemic arterial blood pressure was measured via radial artery catheterization. A Swan-Ganz catheter (Edwards Lifesciences LLC, USA) was inserted for determining central venous pressure, pulmonary capillary pressure, and the cardiac index. Hemodynamic parameters were periodically monitored for a period of 24 h after surgery to detect and adjust arterial blood pressure and the cardiac index to be within 20% of that found at baseline (pre-operation level). Cardioscopy, pulse oximetry, expired CO_2_ levels, nasopharynx temperature, and the BIS index were monitored. The surgery was conducted under mini-CPB and mild hypothermia (34-35°C). Blood samples were collected to measure arterial gases, hemodilution, and electrolytes.

For determination of biochemical and inflammatory markers, arterial blood was sampled at radial catheterization before induction of anesthesia (baseline), 90 min after starting mini-CPB (during surgery), 5 h after starting mini-CPB (within 2-3 h after the end of surgery), and 24 h after the end of surgery.

Cytokines (IL-1, IL-6, IL-10, TNF-α, and INF-γ) were measured by chemical analysis (commercial kits: eBioscience¯, USA). Plasma levels of C-reactive protein were measured by immunoassay (Dimension¯; Siemens, Healthcare Diagnostics Inc., USA). We used a chemiluminescent method (Immulite¯; Siemens, Healthcare Diagnostics Inc.) for determination of cardiac troponin I levels. Creatine phosphokinase (CPK) and CPK-MB levels were measured by enzymatic methods (Dimension¯; Siemens, Healthcare Diagnostics Inc.), cortisol levels were determined using a chemiluminescent enzyme immunoassay (Immulite¯; Siemens, Healthcare Diagnostics Inc.), and glucose levels were measured by a biochromatic method (Dimension¯; Siemens, Healthcare Diagnostics Inc.).

TBARS (20) and ALA-D activity (21) in erythrocytes were determined as described previously.

Plasma samples were coded, and the investigators were blind regarding the treatment regimen. Similarly, all hemodynamic data were collected by trained personnel who were not authors of this study and blind to the anesthetic regimen used. They also recorded the duration of the surgery, the duration of mini-CPB, time for extubation, time in the ICU, and time for in-hospital stay. Postoperative complications and necessity of inotropic support with dobutamine, dopamine, and noradrenaline (i.e., the use of two or more of these inotropic drugs for hemodynamic stability) were recorded. However, their use was necessary only in three patients in the TIVA group and in two in the TIVA+DEX group.

All continuous data are reported as means±SD. Statistical analysis was performed by two-way ANOVA (two anesthetic procedures × four sampling times) with time and treatment as the two factors. Values were considered to be statistically significant when P was <0.05.

## Results

The characteristics of the two groups were similar regarding age, weight, height, comorbidities, mini-CPB time, total surgery time, time for extubation, time in the ICU, and in-hospital stay time (Table 1). Mean arterial pressure was decreased (P<0.01) and heart rate was increased (P<0.01) in a similar manner in the two anesthetic groups after surgery compared with baseline (Supplementary Figure S1). Hemodilution was similar in both anesthetic groups. The hematocrit was significantly decreased after surgery and then increased in the 24-h recovery period after surgery (two-way ANOVA, P<0.0001; Supplementary Figure S1).

**Table:**
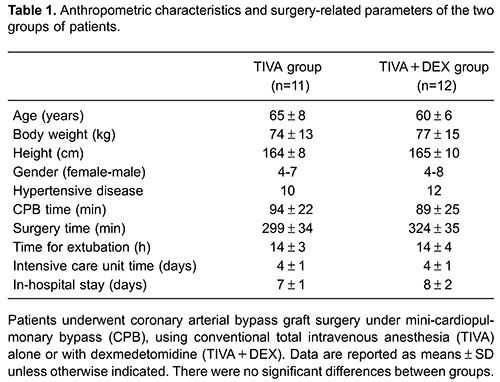

**Figure 1:**
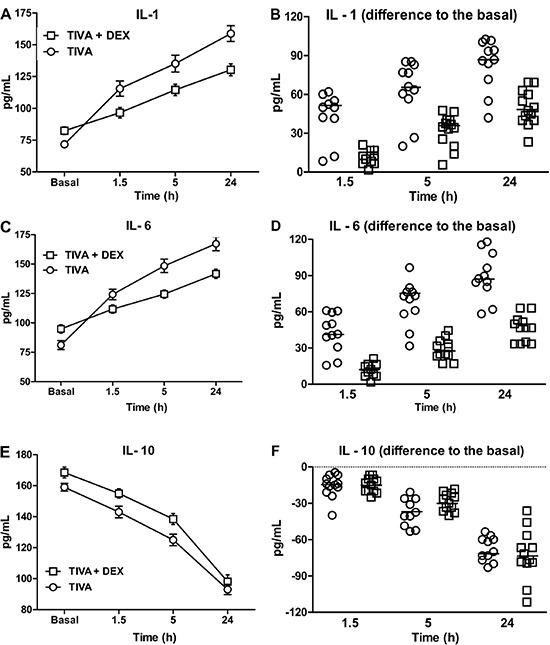
Interleukin (IL)-1 (A,B), IL-6 (C,D), and IL-10 (E,F) levels were measured in patients who underwent coronary arterial bypass graft surgery under mini-cardiopulmonary bypass, using two different types of anesthesia (conventional total intravenous anesthesia [TIVA; n=11] and TIVA+DEX [n=12]). Two-way ANOVA showed a significant interaction between type of anesthesia and sampling time for IL-1 and IL-6 levels (P<0.0001). This finding indicated that the increase in IL-1 and IL-6 levels as a function of time after surgery was attenuated in patients who were anesthetized with TIVA+DEX compared with those who received only TIVA. Two-way ANOVA of IL-10 levels only showed a significant main effect of sampling time (P<0.0001) with a similar and progressive decrease of IL-10 in both anesthetic groups.

Plasma IL-1 (Figure 1A and B) and IL-6 (Figure 1C and D) levels were increased after surgery compared with baseline, but this increase was proportionally higher in the TIVA group compared with the TIVA+DEX group (both P<0.0001). Plasma IL-10 levels were decreased after CABG surgery compared with baseline (P<0.0001; Figure 1E and F). Anesthesia with TIVA or TIVA+DEX did not modify the rate of decrease in IL-10 levels (Figure 1E and F).

**Figure 2:**
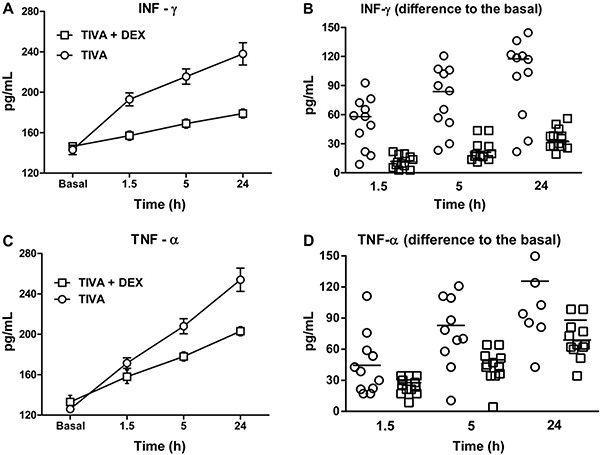
*A, B,* Plasma γ-interferon (INF-γ) and *C*, *D*, tumor necrosis factor-α (TNF-α) levels in patients who underwent coronary arterial bypass graft surgery under mini-cardiopulmonary bypass, using two different types of anesthesia (conventional total intravenous anesthesia [TIVA; n=11] and TIVA+DEX [n=12]). Two-way ANOVA showed a significant type of anesthesia versus sampling time interaction for INF-γ and TNF-α levels (P<0.0001). This finding indicated that the increase in INF-γ and TNF-α levels after surgery was lower in patients who were anesthetized with TIVA+DEX than in patients who received only TIVA.

Plasma INF-γ levels (Figure 2A and B) and TNF-α levels (Figure 2C and D) were increased after surgery compared with baseline. However, the increase in INF-γ and TNF-α levels was proportionally higher in the TIVA group than in the TIVA+DEX group (Figure 2A-D; both P<0.0001).

Plasma C-reactive protein levels were significantly increased only after 24 h (Supplementary Figure S2; P<0.0001), CPK levels gradually increased after surgery (Supplementary Figure S2C and D), and CPK-MB levels immediately increased after the end of surgery compared with baseline (Supplementary Figure S2E and F). However, the changes in these markers were similar between the two anesthetic groups. Troponin, cortisol, and glucose levels were increased after surgery compared with baseline, but no differences were observed between the two anesthetic procedures (Supplementary Figure S3).

**Figure 3:**
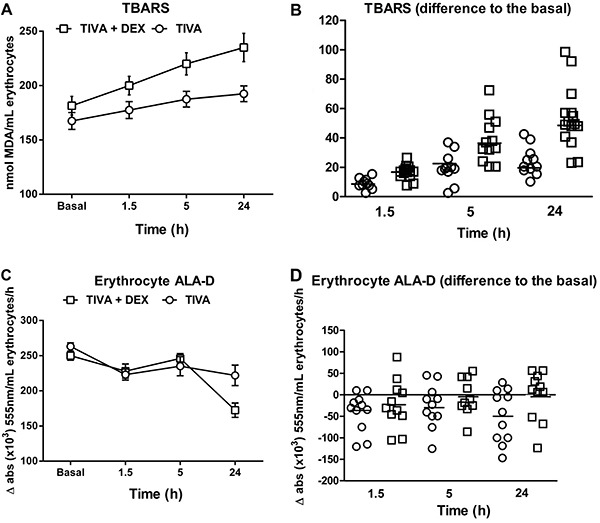
*A*, *B*, Thiobarbituric reactive substances (TBARS) and *C*, *D*, δ-aminolevulinate dehydratase (ALA-D) enzyme activity of erythrocytes in patients who underwent coronary arterial bypass graft surgery under mini-cardiopulmonary bypass, using two different types of anesthesia (conventional total intravenous anesthesia [TIVA; n=11] and TIVA+DEX [n=12]). For TBARS, two-way ANOVA showed a significant sampling time versus type or anesthesia interaction (P<0.001). For ALA-D activity, two-way ANOVA showed only a significant main effect of time (P<0.0001).

TBARS in erythrocytes were increased after surgery compared with baseline. This increase in TBARS was proportionally higher in the TIVA+DEX group than in the TIVA group (Figure 3A and B; P<0.01). ALA-D activity was decreased after surgery compared with baseline, and this decrease was lowest 24 h after surgery in both experimental groups (Figure 3C and D; P<0.001).

## Discussion

The main finding of the present study was that DEX (as a component of TIVA) modified the inflammatory response in CABG surgery under mini-CPB. DEX use was associated with a reduced increase in plasma IL-1, IL-6, TNF-α, and INF-γ levels compared with conventional TIVA. In both groups of patients, a similar postoperative decrease in IL-10 levels occurred. In contrast to our expectation, DEX use was associated with an increase in TBARS levels in erythrocytes after surgery compared with the TIVA group. The decrease in activity of ALA-D, which is considered as a putative marker of oxidative stress (21,22), was similar in both anesthetic groups. Taken together, these results indicated dissociation between changes in the levels of inflammatory markers and oxidative stress in erythrocytes of patients who were subjected to CABG surgery.

There are limited data on the effects of DEX and other α-2 adrenergic receptor agonists on cytokines (13,23 24 25 26 27 28 29 30) and TNF-α production by macrophages (27,28). Taniguchi et al. (25,26) demonstrated that DEX has inhibitory effects on cytokine release triggered by endotoxemia. Therefore, one of the mechanisms of anti-inflammatory effects of DEX may be via modulation of cytokine production by macrophages and monocytes. Similarly, Hofer et al. (29) showed that clonidine decreased proinflammatory cytokine production in experimental sepsis. They observed an improvement in survival after induction of sepsis in mice by preventive administration of clonidine or DEX (29,30). In accordance with our findings, they reported a reduction in the plasma proinflammatory mediators IL-1β, IL-6, and TNF-α (29). They suggested the use of central-acting α2-adrenergic receptor agonists as a preventive therapeutic option in major surgery. In the current study, patients did not have sepsis, but CABG surgery resulted in a high immunological stress response, which to some extent is similar to SIRS. Of particular therapeutic significance, DEX (TIVA+DEX) hampered the surgery-induced increase in IL-1, IL-6, INF-γ, and TNF-α levels compared with conventional TIVA. Notably, one study showed that DEX decreased the inflammatory response in ICU patients with severe sepsis more than did propofol infusion (30), when TNF-α, IL-1, and IL-6 were used as endpoints of inflammation.

Recently, Peng et al. (31) demonstrated that DEX was a potent suppressor of lipopolysaccharide-induced inflammation in activated microglia and may be a potential therapeutic agent for the treatment of intensive care unit delirium. They investigated the effects of DEX on the production of proinflammatory mediators in lipopolysaccharide-stimulated microglia and found that DEX (10 and 100 ng/mL) inhibited the release of nitric oxide, prostaglandin E2, interleukin 1β, and TNF-α. The dosage that we used in the current study can be considered low to moderate. Nevertheless, this dose diminished the surgery-induced increase in plasma IL-1, IL-6, TNF-α, and INF-γ levels.

DEX can inhibit cortisol synthesis, but this has not been reported in short-term use in humans (32 33 34). In fact, Bekker et al. (35) hypothesized that intraoperative administration of DEX reduces the stress response and improves the quality of recovery in patients undergoing major spinal surgery by negatively modulating the increase in plasma cortisol levels observed after surgery (35). However, we did not observe any differences in increased levels of cortisol and glucose between the two anesthetic groups of patients (for details, see Supplementary material).

In this study, we demonstrated that DEX attenuated the increase in IL-1, IL-6, TNF-α, and INF-γ levels in patients under CABG surgery with mini-CPB. One of the limitations of the present study was the small number of enrolled patients. However, the variability in inflammatory markers was satisfactory. Furthermore, the dose of DEX used was low. Future studies on the anti-inflammatory effect of DEX at high doses need to be performed. Indeed, the safety and efficacy of high doses of DEX in patients undergoing major surgeries need to be determined to assess its appropriateness as an ideal anti-inflammatory anesthetic. In short, based on our results, we conclude that DEX is an important anesthetic adjuvant in cardiac surgery.

## Supplementary Material


