# Supplementary material for: Dexmedetomidine decreases the inflammatory response to myocardial surgery under mini-cardiopulmonary bypass
Source: Braz J Med Biol Res. 2016 Feb 23;49(4):e4646. doi: 10.1590/1414-431X20154646 (PMC4792505; doi:10.1590/1414-431X20154646)

Figure S1. *A*, *B*, Mean arterial pressure (MAP); *C*, *D*, heart rate (HR); *E*, *F*, hematocrit (HT) were measured in patients who underwent coronary arterial bypass graft surgery under mini-cardiopulmonary bypass (CPB). Two anesthetic procedures were used: conventional total intravenous anesthesia TIVA (n=11) or TIVA+DEX (n=12). Arterial blood at radial access was collected at four different times. The first sample was before induction of anesthesia (basal). The second sample was collected 90 min after CPB. The third sample was collected 5 h after CPB. The fourth sample was collected 24 h after surgery. Two-way ANOVA showed a significant effect of sampling time (P<0.0001) MAP and HR.

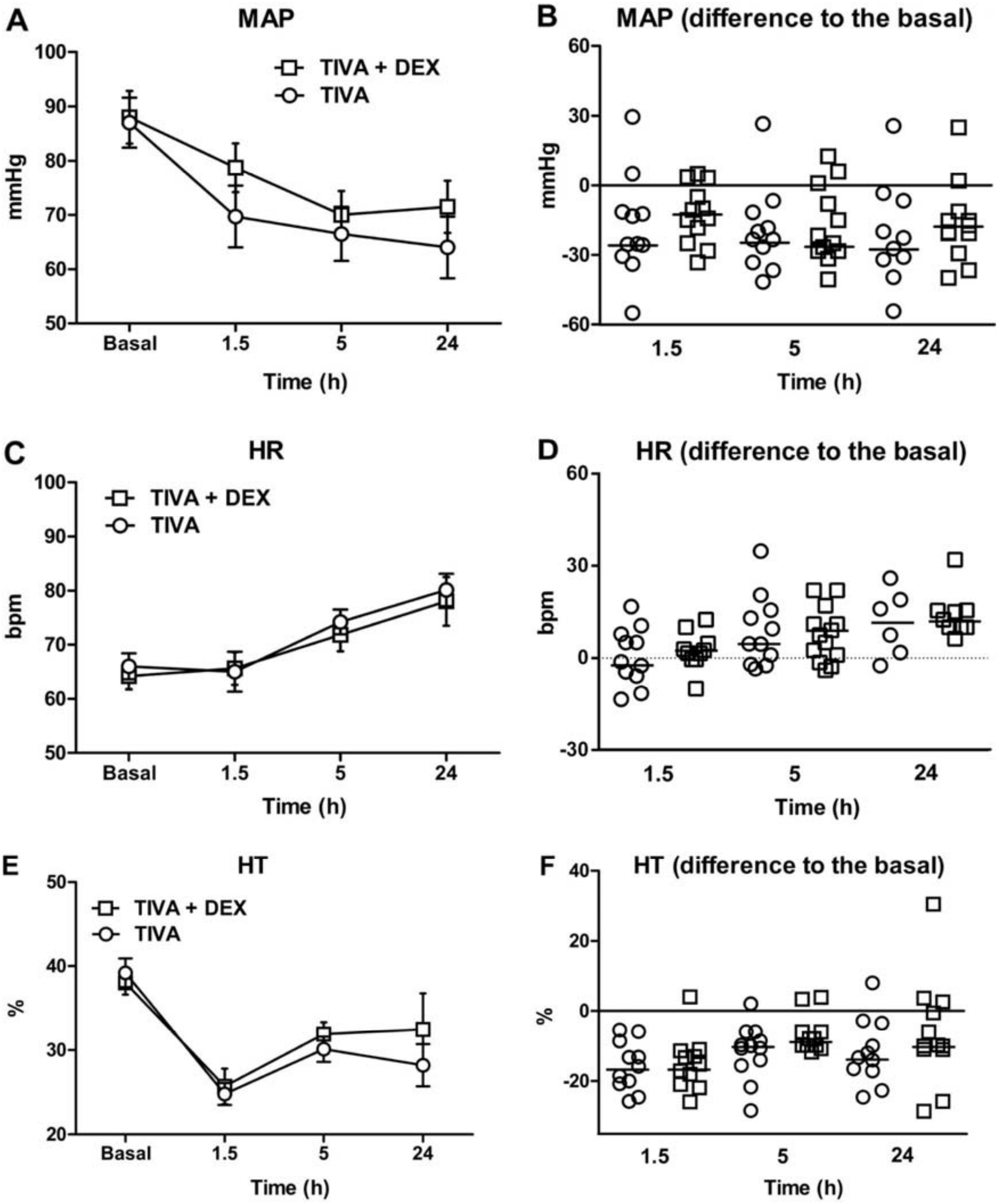

Figure S2. *A*, *B*, Plasma C-reactive protein (CRP); *C*, *D*, creatine phosphokinase (CPK); *E*, *F*, creatine phosphokinase-MB (CPK-MB) were measured in patients who underwent coronary arterial bypass graft surgery under mini-cardiopulmonary bypass. Two anesthetic procedures were used: conventional total intravenous anesthesia TIVA (n=11) or TIVA+DEX (n=12). Two-way ANOVA showed only a significant effect of time ($P<0.0001$) with a significant increase in CRP, CPK, and CPK-MB levels in the two groups as a function of time after surgery.

.

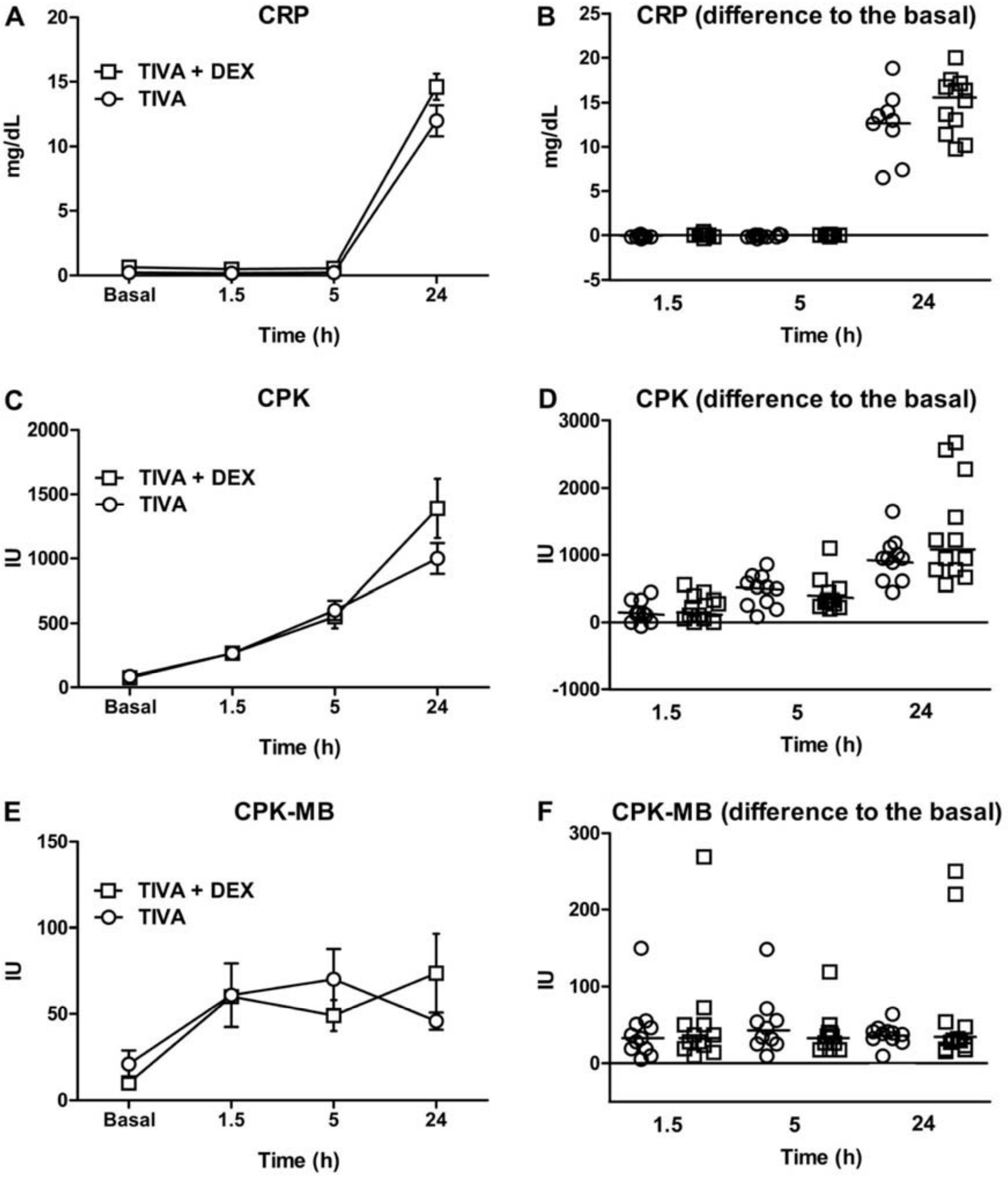

Figure S3. *A*, *B*, Plasma cardiac troponin I (cTn-I) levels; *C*, *D*, cortisol levels; *E*, *F* glucose levels were measured in patients who underwent coronary arterial bypass graft surgery under mini-cardiopulmonary bypass. Two anesthetic procedures were used: conventional total intravenous anesthesia TIVA (n=11) or TIVA+DEX (n=12). Two-way ANOVA showed a significant effect of sampling time for cTn-I, cortisol, and glucose levels (P<0.0001).

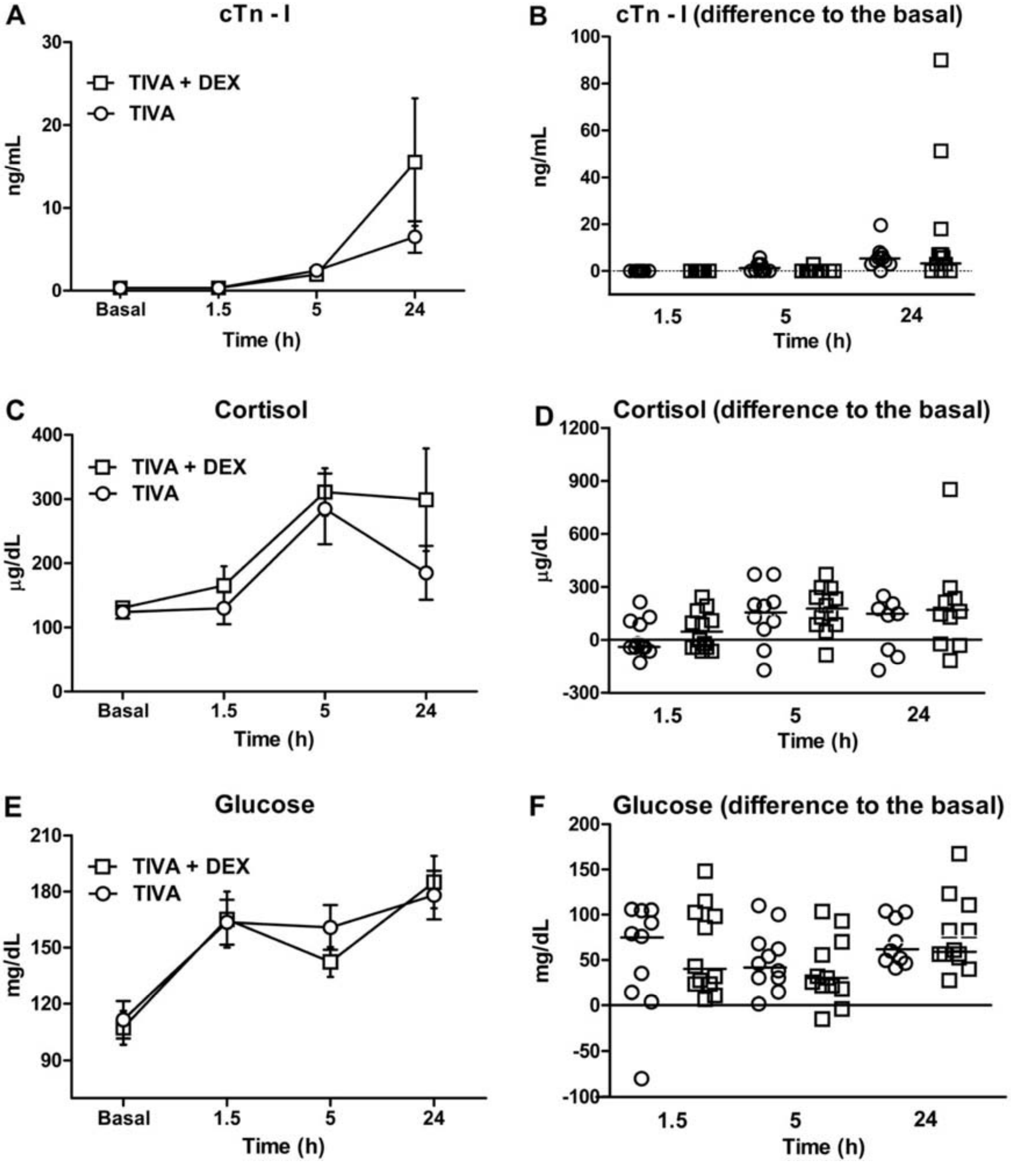

Supplement: Supplementary file 1 [file 1414-431X-bjmbr-1414-431X20154646-S1.pdf]
